# Pain Coping and Healthcare Use in Patients with Early Knee and/or Hip Osteoarthritis: 10-Year Follow-Up Data from the Cohort Hip and Cohort Knee (CHECK) Study

**DOI:** 10.3390/jcm12237455

**Published:** 2023-12-01

**Authors:** Meike C. van Scherpenseel, Corelien J. J. Kloek, Cindy Veenhof, Martijn F. Pisters

**Affiliations:** 1 Research Group Innovation of Human Movement Care, Research Center Healthy and Sustainable Living, HU University of Applied Sciences Utrecht, Heidelberglaan 7, P.O. Box 12011, 3501 AA Utrecht, The Netherlands; corelien.kloek@hu.nl (C.J.J.K.); cindy.veenhof@hu.nl (C.V.); 2 Center for Physical Therapy Research and Innovation in Primary Care, Leidsche Rijn Julius Health Care Centers, 3454 PV De Meern, The Netherlands; m.f.pisters@umcutrecht.nl; 3 Physical Therapy Research, Department of Rehabilitation, Physical Therapy Science and Sport, UMC Utrecht Brain Center, Utrecht University, 3508 GA Utrecht, The Netherlands; 4 Physical Therapy Sciences, Program in Clinical Health Sciences, University Medical Center Utrecht, Utrecht University, 3508 GA Utrecht, The Netherlands; 5 Research Group Empowering Healthy Behavior, Department of Health Innovation and Technology, Fontys University of Applied Sciences, 5600 AH Eindhoven, The Netherlands

**Keywords:** osteoarthritis, healthcare utilization, pain coping, CHECK

## Abstract

Background: Knee and hip osteoarthritis (OA) among older adults account for substantial disability and extensive healthcare use. Effective pain coping strategies help to deal with OA. This study aims to determine the long-term relationship between pain coping style and the course of healthcare use in patients with knee and/or hip OA over 10 years. Methods: Baseline and 10-year follow-up data of 861 Dutch participants with early knee and/or hip OA from the Cohort Hip and Cohort Knee (CHECK) cohort were used. The amount of healthcare use (HCU) and pain coping style were measured. Generalized Estimating Equations were used, adjusted for relevant confounders. Results: At baseline, 86.5% of the patients had an active pain coping style. Having an active pain coping style was significantly (*p* = 0.022) associated with an increase of 16.5% (95% CI, 2.0–32.7) in the number of used healthcare services over 10 years. Conclusion: Patients with early knee and/or hip OA with an active pain coping style use significantly more different healthcare services over 10 years, as opposed to those with a passive pain coping style. Further research should focus on altered treatment (e.g., focus on self-management) in patients with an active coping style, to reduce HCU.

## 1. Introduction

Knee and hip osteoarthritis (OA) are among the most common chronic joint conditions worldwide among older adults [1]. This prevalence is expected to increase due to the growing presence of OA-related risk factors worldwide, such as higher age, obesity, and a sedentary lifestyle [2,3]. Individuals with knee and hip OA experience pain, physical disability, and stiffness [4], and pain is often the key symptom in the decision to seek medical help [5]. Research has shown that patients with OA use more healthcare services than patients without OA, indicating the increased healthcare needs of this population [2,6]. However, the provision of healthcare services is currently focused on the treatment of established OA with already advanced joint damage, with resultant altered joint biomechanics, and often increased chronic pain levels [7]. This makes effective treatment difficult. It has been widely recognized that identifying OA in its earliest stages is important, as subsequent early intervention allows for preventing or slowing the progression of structural destruction of the joint, and simultaneously improving long-term outcomes [7,8].

Patients with knee or hip OA are predominantly managed in primary care, e.g., by using analgesics and consulting general practitioners [9]. Other advanced treatment modalities in secondary care, such as consultation with a rheumatologist or performing surgery, are used less often [9]. This is in line with the Stepped Care Strategy to manage care in patients with OA, described in recent Dutch treatment guidelines [10]. The Stepped Care Strategy presents the optimal order to employ treatment options, recommending to consider advanced treatment modalities only if the options in the previous steps failed to lead to satisfactory results [11]. Most current treatment options for OA focus on reducing pain and functional limitations and improving health-related quality of life (HRQoL) [10]. Aiming at patients’ HRQoL is essential, since patients with musculoskeletal chronic diseases, such as OA, report among the lowest HRQoL [12]. To improve HRQoL and well-being in chronic pain patients, it is essential to evaluate and promote patients’ coping strategies [13].

Pain coping strategies are cognitive and behavioral reactions to chronic pain to manage the pain [14,15,16]. Pain coping strategies are commonly categorized as active or passive [17]. People with an active, or adaptive, coping style aim to self-manage the pain or attempt to function despite the pain [17]. This has been shown to lead to a more functional lifestyle and less pain [18,19]. People with a passive, or maladaptive, coping style tend to avoid the pain or relinquish the control of their pain to others [17]. This is associated with higher levels of pain, greater functional disability, and a reduction in HRQoL [15,17,20]. Research has shown that people are prone to use one type of coping over another and this is influenced by many factors, such as pain intensity, illness experience, attitude towards disease, and trust in medical help [14,21,22]. Without intervening, coping style seems to remain fairly stable over time in patients with OA [15,23].

Since it is known that having a passive pain coping style is related to higher levels of pain, it may be assumed that these patients would seek more help in healthcare to cope with their limitations. However, prior research concluded that having an active pain coping style is a significant predictor of high healthcare use (HCU) in patients with OA at 2 years [9,24]. It is suggested that patients with an active pain coping style intend to step out of the role of ‘passive sufferer’ and become a more active, self-actualizing individual by seeking help [21]. Therefore, it may be expected that they develop skills to cope with their disease independently in the first years of OA. This may lead to less utilization of healthcare in the long term.

However, it is currently unknown how pain coping style and HCU are related in the long term in patients with OA. Research examining this relationship in the long term is of special interest, given the chronically painful and incurable nature of OA. Therefore, this study aimed to determine the long-term relationship between pain coping style in patients with knee and/or hip OA in an early stage and the course of healthcare use over a follow-up period of 10 years.

## 2. Materials and Methods

### 2.1. Design

To determine the relationship between pain coping style and HCU in the long term, baseline and annually measured data for 10 years from the Cohort Hip and Cohort Knee (CHECK) cohort were used [25]. CHECK was a prospective longitudinal multicenter cohort study with 1002 participants with early symptomatic knee and/or hip OA in the Netherlands. The CHECK cohort was approved by the medical ethics committees of all participating centers (METC 02-017), and all participants gave their written informed consent. To ascertain adequate presentation of this observational study, The Strengthening the Reporting of Observational studies in Epidemiology (STROBE) guidelines were followed [26] (Table S1).

### 2.2. Setting and Study Population

Participants throughout the Netherlands were included in the CHECK cohort through convenience sampling. Participants who visited their general practitioner and potentially met the inclusion criteria were sent to one of the ten participating hospitals, where final eligibility was determined by a physician. Inclusion criteria were (1) having pain of the knee and/or hip; (2) age between 45 and 65 years, and (3) being at or within 6 months of first contact with the general practitioner for symptoms. Participants were excluded if they met any of the following exclusion criteria: (1) knee and/or hip pain was based on any other pathological condition that could explain the symptoms; (2) comorbidity precluding physical evaluation and/or follow up of at least 10 years; (3) malignancy in the past 5 years; and (4) inability to understand the Dutch language.

Of the included participants in the CHECK cohort, two groups were formed at baseline by the coordinating study group based on their presenting symptoms: a variable visiting group (participants with mild symptoms) and an annual visiting group (participants with more serious symptoms) [25]. Participants with ‘more serious symptoms’ fulfilled the clinical American College of Rheumatology (ACR) criteria for the classification of knee and/or hip OA [27,28]. For the current study, participants with ‘more serious symptoms’ were included. Participants with ‘more serious symptoms’ can be classified as being in an early phase of the disease, since clinical characteristics such as pain, stiffness, and disability are more prominent in the early phase of OA, and not yet accompanied by radiographic changes related to OA [29]. In the following phase, patients are coping with pain and physical limitations, which leads to a decrease in reporting these clinical characteristics, while structural changes in the joint develop. In other words, the recruitment of patients in an early phase of OA may carry more perceived symptoms of OA than in a later stage of the disease [29]. Therefore, the CHECK cohort can be recognized as an “early” symptomatic knee and/or hip cohort [29].

Participants were followed for a total period of 10 years, starting between 2002 and 2005. Participants visited the centers annually. Study visits consisted of structured interviews, self-reported questionnaires, physical examinations, X-rays, and blood and urine collection [25]. Data from self-reported questionnaires, measuring pain, health status, and quality of life, were used for the current study.

### 2.3. Measurement Instruments

#### Main Study Parameters

HCU was measured annually using a combined version of a self-reported questionnaire, developed for the Patient Panel Chronic Diseases by Nivel (The Netherlands Institute for Health Services Research) [30], and the questionnaire Economic Aspects in Rheumatoid Arthritis [31]. In the HCU questionnaire, all available OA-related healthcare services were included (range 0–20), for example, contact with the general practitioner and medical specialist. Also, medical use was included, such as the use of nonsteroidal anti-inflammatory drugs (NSAIDs). In this study, the term ‘services’ was used to refer to all available healthcare options in the HCU questionnaire. At each study visit, participants reported whether or not (yes/no) they had used the healthcare service(s) in the past 3 months. For the use of medication, the participant indicated whether or not they were using this (yes/no) at the moment. The participants did not need to specify how many times they had used the service(s). Per participant, this ultimately led to a range of 0 to 20 used service(s) in the past 3 months. Score 0 represents “no usage of healthcare services” and score 20 represents “usage of all available healthcare services”.

At baseline, pain coping was identified with the Pain Coping Inventory (PCI) [16]. The PCI is a self-reported questionnaire that determines whether a person has an active or passive pain coping style. The PCI has 33 items, divided over six subscales. The active coping subscales were defined as the following three active strategies: pain transformation, distraction, and reducing demands. Passive coping subscales were defined as the three passive strategies: retreating, worrying, and resting [16]. To interpret the score, the points given on the three active and passive subscales were added up and divided by the maximum points of the active and passive subscales, respectively. This led to a percentage. The subscale with the largest percentage determined which pain coping style was used the most by the individual [16].

### 2.4. Other Study Parameters

The following patient characteristics, and potential confounders, were administered at baseline: age, gender, body mass index (BMI), educational level, employment, number of comorbidities (range 0–4), and location of OA [25]. Various disease-related factors might interfere with the association between pain coping and HCU and were therefore evaluated. Patient-reported outcomes were measured using self-reported questionnaires. Pain intensity was measured with the Numeric Rating Scale (NRS), an 11-point unidimensional pain rating scale (0–10) [32]. A score of 0 represents “no pain” and a score of 10 represents “worst pain imaginable”. The Western Ontario McMasters University Osteoarthritis Index (WOMAC) was used to evaluate condition-specific health status [33]. WOMAC assesses three dimensions: pain (0–20), functioning (0–68), and stiffness (0–8). WOMAC scores were standardized, leading to all subscales showing scores between 0 and 100. Higher scores indicate worse pain, functional limitations, and stiffness. Self-reported HRQoL was measured with the Short Form (SF-)36 [34]. The questionnaire has a score range of 0 to 100, with higher scores indicating a better HRQoL. For an overview of all measures that were included in the CHECK cohort, but have not been incorporated in the current study, see Wesseling et al. (2016) [25].

### 2.5. Statistical Analysis

Statistical analyses were performed using IBM SPSS Statistics^®^, version 25.0. The data were checked for data entry errors, outliers, and missing data. To indicate whether values were missing completely at random (MCAR) or missing at random (MAR), significant differences in baseline characteristics between participants with and without missing values at baseline were tested using independent T-tests for continuous values and chi-squared tests for categorical values. If significantly different, values were considered MAR, and imputation was conducted to reduce bias [35]. Multiple imputation with fully conditional specification was used [36]. A total of 10 different imputed datasets were generated. Both categorical and continuous variables went into the imputation algorithm. Ultimately, the imputed sets of parameter estimates were pooled using Rubin’s rules of combination [37]. A sensitivity analysis was conducted to examine assumptions of the missing data by analyzing complete cases only [38]. Descriptive statistics were used to analyze baseline characteristics of the imputed data and the mean of used healthcare services and to calculate average follow-up time. Also, the change in pain intensity and health status over time was described by using descriptive statistics, to gain more insight into the deterioration or improvement of these factors in both groups. For a further analysis, the twenty healthcare services were clustered into five subgroups by the research team (M.S., C.K., M.P.). The classification of the subgroups was based on the Stepped Care Strategy [39], to take into account the stepwise progression in advanced treatment modalities in the management of knee and/or hip OA [10,24]: (1) self-care—e.g., use of paracetamol, family/household help; (2) NSAIDs—e.g., use of diclofenac, naproxen; (3) primary care—e.g., contact with a general practitioner, physiotherapist; (4) secondary care—e.g., contact with an orthopedist, rheumatologist; (5) work-related care—e.g., company doctor (Table S2).

To determine the relationship between pain coping style and the course of the number of healthcare services used over 10 years, a longitudinal analysis was performed. To estimate the average relationship over the entire population, a General Estimating Equation (GEE) analysis was used. GEE takes into account the dependency of individual observations by specifying a working correlation structure [35]. The literature has shown that HCU may change over time [9,24] and therefore, an unstructured correlation structure was chosen. Since pain coping style is a dichotomous variable, PCI at baseline was dummy-coded. HCU is a count variable and therefore it follows a Poisson distribution. Therefore, initially, a Poisson regression model was proposed. However, assumptions for Poisson regression were checked and overdispersion was present in the data [40]. To take overdispersion into account, a negative-binomial regression model was used [40]. Furthermore, baseline characteristics and disease-related variables were tested for interference with the association between PCI and HCU. A 10% cut-off for change-in-estimate was used to identify confounders [41]. Eventually, the relationship between PCI and HCU was adjusted for confounders. Furthermore, a secondary analysis, adjusted for confounders, was performed to gain insight into which subgroups of HCU were visited the most. Significance of all tests was defined at the level of *p* ≤ 0.05.

## 3. Results

Of the 1002 participants included in the CHECK cohort, 861 met the inclusion criteria for the current study. A total of 120 participants dropped out during the study. The reason for dropping out was unknown. The average follow-up time was 9.3 years. At baseline, PCI subscales had between 2.6% and 2.9% missing values. Over the years, HCU had missing data of 2.1% at baseline to 15% at T10. Of all values, 8.8% were missing and tests showed that the values were MAR. Therefore, multiple imputation was performed. Participants with missing values were significantly more often female (*p* = 0.04) and had a higher score on the WOMAC subscale physical functioning (*p =* 0.017).

Baseline characteristics of the study population are presented in Table 1. The majority of the participants had both knee and hip OA (47.5%), one or more comorbidities (7%), and an active pain coping style (86.5%). For a more detailed description of the total population of the CHECK cohort, see Wesseling et al. (2016) [25].

### 3.1. Change in Pain Intensity and Health Status

The level of pain intensity and scores of the WOMAC subscales remained stable between baseline and 10-year follow up (Table 2). Pain intensity ‘right now’ and ‘past week’ scores remained relatively unchanged in patients with an active pain coping style and decreased in patients with a passive pain coping style. All WOMAC subscale scores decreased in both patients with an active and passive pain coping style.

### 3.2. Course of Healthcare Use

**Figure 1:**
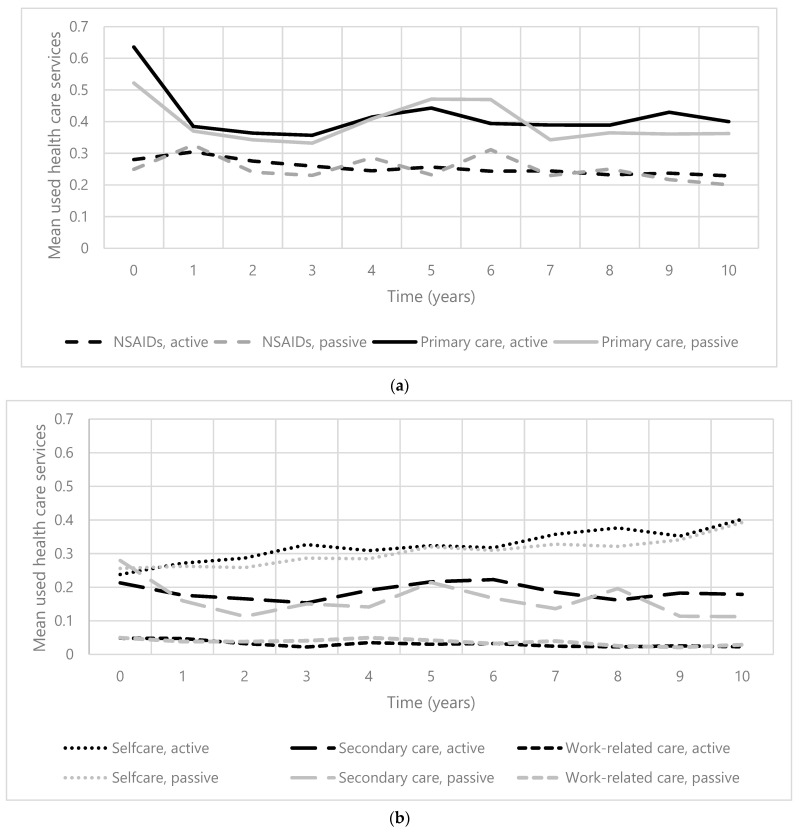
(**a**). The course of mean used healthcare services (0–20) in primary care and NSAIDs over 10 years in patients with early knee and/or hip OA. Abbreviation: NSAIDs = nonsteroidal anti-inflammatory drugs. (**b**). The course of mean used healthcare services (0–20) in self-care, secondary care, and work-related care over 10 years in patients with early knee and/or hip OA.

Over 10 years, the mean of all used healthcare services (range 0–20) in participants with an active pain coping style ranged from 1.18 (±1.75) to 1.42 (±1.49), and in participants with a passive pain coping style, from 0.99 (±1.57) to 1.35 (±1.80). Figure 1a,b present the change in the mean number of used healthcare services in all subgroups, for both participants with an active and passive pain coping style. The mean number of used healthcare services was the highest in the subgroup ‘primary care’ in all participants. Used healthcare services in the subgroup ‘self-care’ increased over time, in contrast to the use of NSAIDs, which slightly decreased in both patients with an active and passive coping style. The trajectories of HCU in subgroups ‘primary care’ and ‘secondary care’ over the years are comparable. The course of mean used healthcare services in the subgroup ‘work-related care’ remains relatively stable in both patients with an active and passive coping style.

### 3.3. Relationship Pain Coping Style and HCU

The analysis showed that the following variables interfered with the relationship between pain coping style and the course of the number of used healthcare services over 10 years: location of OA, NRS pain now and NRS pain past week, all WOMAC subscales, and all subscales of the SF-36, except the subscale of general health. The results of the GEE analysis on the relationship between pain coping style in an early stage of OA and the course of the number of used healthcare services over 10 years, unadjusted and adjusted for confounders, are shown in Table 3. Having an active pain coping style is statistically significantly (*p* = 0.022) associated with an increase of 16.5% (95% CI, 2.0–32.7) in the number of the use of different healthcare services over 10 years when adjusted for confounders. The sensitivity analysis showed no remarkable differences between imputed data and original data.

The secondary analysis (Table 4), adjusted for confounders, showed that having an active coping style was significantly (*p* = 0.037) associated with an increase of 18.4% (95% CI, 1.1–38.7) in used healthcare services in primary care. Pain coping style was not significantly associated with subgroups secondary care (*p* = 0.117), self-care (*p* = 0.118), NSAIDs (*p* = 0.324), and work-related care (*p* = 0.920).

## 4. Discussion

This study aimed to examine the relationship between pain coping style in an early stage of OA and healthcare use over 10 years in patients with knee and/or hip OA. Results showed that patients with an active pain coping style use more different healthcare services over 10 years, compared to patients having a passive pain coping style. These results are independent of the change in pain and functioning over time as we adjusted for these confounders.

To our knowledge, no prior studies examined the relationship between pain coping style and HCU in patients with any chronic musculoskeletal conditions over 10 years. However, there have been studies that examined factors that predict HCU in patients with knee and/or hip OA at 2 years. Hoogeboom et al. [9] (2012) found that having an active pain coping style was a risk factor for analgesic use. They also used data from the CHECK cohort. Smink et al. (2014) [24] identified that an active pain coping style was a determinant for the use of more than one healthcare modality. These results correspond with the results in the current study. As also hypothesized by these researchers [24], these findings may be explained by the fact that people with an active pain coping style intend to dissociate themselves as a ‘passive sufferer’ and start acting as self-actualizing, active individuals. In this latter role, they may seek multiple types of help in the healthcare domain to become able to actively deal with their OA-related complaints. However, several OA guidelines recommend self-management interventions as a core component in the management of OA, which may help patients with an active pain coping style to deal with OA independently [10,42]. Self-management is defined as the individual’s ability to cope effectively with the disease, symptoms, treatment, psychosocial and physical consequences, and changes in lifestyle inherent to living with OA [43]. Critical components of self-management include education, physical activity, and weight management [44]. Self-management interventions have the potential to improve pain, physical function, joint function, and quality of life in patients with knee OA [45,46,47]. Consequently, self-management as a component of treatment leads to reduced HCU and accompanied healthcare costs, since patients require less help from healthcare professionals [48,49,50,51,52]. Unfortunately, due to the nature of the CHECK data, it was unknown whether healthcare professionals in the CHECK cohort promoted self-management.

From the perspective of prevention and early intervention, a diagnosis of early OA is essential to prevent or delay the progression of OA before irreversible destruction of the joint occurs [7,8]. The CHECK cohort has been addressing a major gap in this area, as they initiated a cohort with “early” symptomatic OA of the knee and/or hip [25]. However, to date, the concept and diagnostic criteria of “early OA” are mostly dependent on expert opinions and lack higher levels of evidence [8]. Currently suggested criteria of early OA include, among other things, self-reported pain and other symptoms such as function, radiological findings, and clinical examination [7,8]. Detailed classification, definition, and validation of “early OA” are necessary to allow the optimal management of the disease with long-term benefits, before progressive and irreversible changes of the joint occur [7,8].

A surprising characteristic of the participants in the CHECK cohort was the large percentage (86.5%) of people with an active pain coping style at baseline. This may be due to several reasons. First, potential participants were excluded when they did not understand the Dutch language, whereas individuals with a non-Western background living with chronic pain and struggling with language barriers often use passive pain coping strategies [53]. This may have led to a biased sample, which may have affected the external validity of the study. Second, the majority of the participants were female and previous research has shown that women are more likely to use active coping styles [54].

Furthermore, results showed that active copers in this study did not show clinically relevant improvement in health outcomes in comparison with passive copers over time [55]. These results are remarkable, since we initially expected to observe better physical health outcomes in people with an active pain coping style, since this trend is frequently presented in prior research [10]. However, we did not examine these results in detail and therefore, we cannot make any firm statements regarding these scores.

An interesting finding in our study is that primary care services were used the most of all healthcare services. This is a positive finding since this is in line with the recommendations according to the Stepped Care Strategy in the management of OA [11]. In addition, the upward trend of used self-care and the downward trend of used secondary care are also in line with stepped care.

Strengths of this study include the large sample size of the CHECK cohort and the participation of ten different general and university hospitals throughout the Netherlands, which generated a rich and nationally representative data sample. Also, there was only a small amount of missing data, except from HCU at the last time point. Multiple imputation was used to reduce bias. Furthermore, the data were gathered over a long period, which is suitable for the long-lasting condition of OA and resulted in the opportunity to find patterns in HCU that occurred over the period.

There are also some limitations to this study. First, the HCU questionnaire only determined whether or not participants used prespecified healthcare services, and not how many times they used the services. Consequently, we were not able to present detailed data on the specific amount of utilized healthcare services. Second, self-reported HCU in patients with OA is often underreported, providing no accurate information [56]. For future cohort studies, we recommend measuring HCU over the entire period and including the volume as well. To achieve an accurate and unbiased representation of HCU, retrospective cost diaries [57] in combination with patients’ medical files [24,58] or administrative databases of healthcare insurances can be used [2]. Third, the confidence intervals of the results are wide, despite the large sample. This suggests a high dispersion in the data and represents the uncertainty in a generalization. Nevertheless, data show us results we should not ignore, since they indicate a potentially large impact on the healthcare system. In addition, these results are confirmed in other studies as well. At last, the content of the treatments was unknown. Therefore, we cannot determine which element of a given treatment may have contributed to an increase or reduction in used healthcare services per person.

## 5. Conclusions

In conclusion, the results of this study show that patients with early knee and/or hip OA with an active pain coping style use significantly more different healthcare services over 10 years, as opposed to those with a passive pain coping style. Further research is necessary to examine whether focusing on self-management skills as part of a treatment in people with an active coping style leads to a reduction in the number of used healthcare services in the long term.

## Figures and Tables

**Table 1:** Baseline characteristics of the study population *.

Characteristics at Baseline	All Participants	Participants with an Active Pain Coping Style	Participants with a Passive Pain Coping Style
Number (%)	861	745 (86.5)	116 (13.5)
Age, mean (SD)	56 (5.2)	56 (5.3)	56 (5.0)
Sex, female (%)	81.1%	81.2%	79.9%
BMI, median (IQR)	26 (24–28)	26 (23–28)	26 (24–29)
Location OA			
Hip	15.3%	15.6%	12.8%
Knee	37.2%	36.2%	43.5%
Knee and hip	47.5%	48.1%	43.7%
Comorbidities			
0	25.9%	26.2%	24.0%
1	30.2%	30.0%	31.7%
2	21.4%	22.0%	17.6%
3	12.7%	12.3%	15.3%
≥4	9.8%	9.5%	11.4%
Married/partnership, yes	83.0%	83.5%	79.6%
Level of education			
No/primary school	20.1%	19.6%	23.5%
Secondary (vocational) education	46.9%	47.7%	42.2%
Higher education/university	33.0%	32.7%	34.3%
Employed, yes	39.9%	40.6%	35.8%
Pain intensity (0–10), mean (SD)			
Right now	3.2 (2.1)	3.2 (2.0)	3.0 (2.2)
Past week	3.7 (2.1)	3.7 (2.1)	3.5 (2.1)
WOMAC subscales standardized (0–100), median (IQR)			
Pain	25 (15–35)	25 (15–35)	25 (10–40)
Stiffness	37.5 (25–50)	37.5 (25–50)	37.5 (12.5–50)
Function	22.1 (11.8–35.3)	22.1 (11.8–35.3)	22.1 (13.2–36.8)

* Data based on the multiple imputed dataset. Abbreviations: SD = standard deviation; BMI = body mass index; IQR = interquartile range; WOMAC = Western Ontario McMasters University Osteoarthritis Index.

**Table 2:** Difference in scores of pain intensity and health status between baseline and 10 years for patients with an active and passive pain coping style.

	T10		Δ T0–T10	
	Active Pain Coping Style	Passive Pain Coping Style	Active Pain Coping Style	Passive Pain Coping Style
Pain intensity (0–10), mean (SD)				
Right now	3.3 (2.4)	2.7 (2.3)	+0.1	−0.3
Past week	3.7 (2.4)	3.2 (2.3)	0	−0.3
WOMAC subscales standardized (0–100), median (IQR)				
Pain	21 (IQR: 10–35)	15 (IQR: 5–35)	−4	−10
Stiffness	25 (IQR: 20–20)	25 (IQR: 12.5–50)	−12.5	−12.5
Function	20.6 (IQR: 8.8–35.3)	19.1 (IQR: 8.8–39.7)	−2.5	−3

Abbreviations: SD = standard deviation; WOMAC = Western Ontario McMasters University Osteoarthritis Index; IQR = interquartile range. Δ T0–T10 = difference between scores at T0 and T10.

**Table 3:** Relationship between pain coping style and the number of used healthcare services over 10 years in patients with early knee and/or hip OA.

	Number of Used Healthcare Services		
Parameters	B [95% CI]	IRR [95% CI]	*p*
Pain coping style (passive = 0; active = 1)	0.080 [−0.061; 0.329]	1.083 [0.940; 1.390]	0.453
Pain coping style * (passive = 0; active = 1)	0.153 [0.022; 0.283]	1.165 [1.020; 1.327]	0.022

Abbreviations: B = beta (regression coefficient); 95%CI = 95% confidence interval; IRR = incidence rate ratio. * Adjusted for confounders: location OA, NRS pain right now, NRS pain past week, all WOMAC subscales, and all SF36 subscales (except the subscale of general health).

**Table 4:** Secondary analysis on the relationship between pain coping style and the number of used healthcare services over 10 years in patients with early knee and/or hip OA, per subgroup.

	Number of Used Healthcare Services		
Subgroup	B [95% CI]	IRR [95% CI]	*p*
Primary care	0.169 [0.011–0.327]	1.184 [1.011–1.387]	0.037
Secondary care	0.186 [−0.047–0.420]	1.204 [0.954–1.522]	0.117
Self-care	0.170 [−0.043–0.383]	1.185 [0.958–1.467]	0.118
NSAIDs	0.103 [−0.101–0.307]	1.108 [0.904–1.359]	0.324
Work-related care	−0.024 [−0.487–0.440]	0.976 [0.614–1.553]	0.920

Abbreviations: B = beta (regression coefficient); 95%CI = 95% confidence interval; IRR = incidence rate ratio.

## Data Availability

Data is unavailable for sharing as we do not possess ownership of the data. CHECK is the rightful owner of all data that has been used in the current study.

## Supplementary Materials

The following supporting information can be downloaded at: https://www.mdpi.com/article/10.3390/jcm12237455/s1, Table S1. STROBE checklist. Table S2. Subgroups of healthcare use.
