# Supplementary material for: Pain Coping and Healthcare Use in Patients with Early Knee and/or Hip Osteoarthritis: 10-Year Follow-Up Data from the Cohort Hip and Cohort Knee (CHECK) Study
Source: J Clin Med. 2023 Dec 1;12(23):7455. doi: 10.3390/jcm12237455 (PMC10707715; doi:10.3390/jcm12237455)
Supplement: Supplementary file 1 [file jcm-12-07455-s001.zip › jcm-2693405-supplementary.pdf]

**Table S1. STROBE checklist.**

STROBE Statement—checklist of items that should be included in reports of observational studies

**Pain coping and healthcare use in patients with early knee and/or hip osteoarthritis: 10-year follow-up data from the Cohort Hip and Cohort Knee (CHECK) study**

| | Item No. | Recommendation | Page No. | Relevant text from manuscript |
|---|---|---|---|---|
| **Title and abstract** | 1 | (*a*) Indicate the study's design with a commonly used term in the title or the abstract | 1-3 | **Title:** Data from the Cohort Hip and Cohort Knee (CHECK) study<br>**Abstract:** Design—Prospective cohort study |
| | | (*b*) Provide in the abstract an informative and balanced summary of what was done and what was found | 3 | **Abstract:** Methods and Results |
| **Introduction** | | | | |
| Background/rationale | 2 | Explain the scientific background and rationale for the investigation being reported | 4-5 | **Introduction:** Knee and hip osteoarthritis (OA) are among the most common chronic joint conditions worldwide among older adults. This prevalence is expected to increase due to the growing presence of OA-related risk factors worldwide, such as higher age, obesity, and a sedentary lifestyle. Individuals with knee and hip OA experience pain, physical disability, and stiffness. Pain is often the key symptom in the decision to seek medical help [5]. Research showed that patients with OA use more healthcare services than patients without OA, leading to substantial medical costs [...]. Most current treatment options for OA focus on reducing pain and functional limitations and improving health-related quality of life (HRQoL) [...]. To improve HRQoL and well-being in chronic pain patients, it is essential to evaluate and promote patients' coping strategies [...]. Since extensive literature has shown that having a passive pain coping style is related to higher levels of pain, it may be assumed that these patients would seek more healthcare |

| | | | | |
|---|---|---|---|---|
| | | | | services to cope with their limitations. However, prior research concluded that having an active pain coping style is a significant predictor of high healthcare use (HCU) in patients with OA at 2 years [...]. |
| Objectives | 3 | State specific objectives, including any prespecified hypotheses | 4-5 | **Introduction:** It is suggested that patients with an active pain coping style intend to step out of the role of 'passive sufferer' and become a more active, self-actualizing individual by seeking help. Therefore, it may be expected that they develop skills to cope with their disease independently in the first years of OA. This may lead to less utilization of healthcare in the long term. However, it is currently unknown how pain coping style and HCU are related in the long term in patients with OA. Research examining this relationship in the long term is of special interest, given the chronically painful and incurable nature of OA. |
| **Methods** | | | | |
| Study design | 4 | Present key elements of study design early in the paper | 5 | **Materials and Methods, design:** CHECK was a prospective longitudinal multicenter cohort study with 1002 participants with early symptomatic knee and/or hip OA in the Netherlands. |
| Setting | 5 | Describe the setting, locations, and relevant dates, including periods of recruitment, exposure, follow-up, and data collection | 5 | **Materials and Methods, setting, and study population:** Participants throughout the Netherlands were included in the CHECK cohort [...]. Participants who visited their general practitioner and potentially met the inclusion criteria were sent to one of the ten participating hospitals. Final eligibility was determined by a physician at each hospital. Participants were followed for a total period of 10 years, starting between 2002 and 2005. Participants visited the centers annually. Study visits consisted of structured interviews, self-reported questionnaires, physical examinations, X-rays, and blood and urine collection. Data from self-reported questionnaires, measuring pain, health status, and quality of life, were used for the current study. |
| Participants | 6 | (*a*) *Cohort study*—Give the eligibility criteria and the sources and methods of | 5 | **Materials and Methods, setting, and study population:** Participants throughout the Netherlands were included [...] through convenience sampling. Inclusion criteria were (1) having pain of the knee and/or hip; (2) age between 45 |

| | | | | |
|---|---|---|---|---|
| | | selection of participants. Describe methods of follow-up<br><br>*Case-control study*—Give the eligibility criteria and the sources and methods of case ascertainment and control selection. Give the rationale for the choice of cases and controls<br><br>*Cross-sectional study*—Give the eligibility criteria and the sources and methods of selection of participants | | and 65 years, and (3) being at or within 6 months of first contact with the general practitioner for symptoms. Participants were excluded if they met any of the following exclusion criteria: (1) knee and/or hip pain was based on any other pathological condition that could explain the symptoms; (2) comorbidity precluding physical evaluation and/or follow up of at least 10 years; (3) malignancy in the past 5 years; and (4) inability to understand the Dutch language. In the CHECK cohort, two groups were formed: participants with mild symptoms, and those with severe symptoms. For the current study, only participants with 'severe symptoms' were included, because they fulfilled the clinical American College of Rheumatology (ACR) criteria for classification of knee and/or hip OA. Participants who visited their general practitioner and potentially met the inclusion criteria were sent to one of the ten participating hospitals. Final eligibility was determined by a physician at each hospital. |
| | | (*b*) *Cohort study*—For matched studies, give matching criteria and number of exposed and unexposed<br><br>*Case-control study*—For matched studies, give matching criteria and the number of controls per case | N/A | N/A |
| Variables | 7 | Clearly define all outcomes, exposures, predictors, potential confounders, and effect modifiers. Give diagnostic criteria, if applicable | 5-6 | **Materials and Methods, measurement instruments: Main study parameters, other study parameters** |
| Data sources/ measurement | 8* | For each variable of interest, give sources of data and details of methods of assessment (measurement). Describe comparability of assessment methods if there is more than one group | 5-6 | **Materials and Methods, measurement instruments: Main study parameters, other study parameters** |

| | | | | |
|---|---|---|---|---|
| Bias | 9 | Describe any efforts to address potential sources of bias | 7 | **Statistical Analysis:** To indicate whether values were missing completely at random (MCAR) or missing at random (MAR), significant differences in baseline characteristics between participants with and without missing values at baseline were tested using independent T-tests for continuous values and chi-squared tests for categorical values. If significantly different, values were considered MAR, and imputation was conducted to reduce bias [32]. Multiple imputation with fully conditional specification was used [33]. A total of 10 different imputed datasets were generated. |
| Study size | 10 | Explain how the study size was arrived at | 5 | **Materials and Methods, setting, and study population:** For the current study, only participants with 'severe symptoms' were included, because they fulfilled the clinical American College of Rheumatology (ACR) criteria for the classification of knee and/or hip OA. |

Continued on next page

| | | | | |
|---|---|---|---|---|
| Quantitative variables | 11 | Explain how quantitative variables were handled in the analyses. If applicable, describe which groupings were chosen and why | 7 | **Materials and Methods, Statistical Analysis:** Descriptive statistics were used to analyze baseline characteristics of the imputed data and the mean of used healthcare services and to calculate average follow-up time. For a further analysis, the twenty healthcare services were clustered into five subgroups by the research team [...]. The classification of the subgroups was based on the SCS, to take into account the stepwise progression in advanced treatment modalities in the management of knee and/or hip OA [...]. Since pain coping style is a dichotomous variable, PCI at baseline was dummy-coded. |
| Statistical methods | 12 | (*a*) Describe all statistical methods, including those used to control for confounding | 7-8 | **Materials and Methods, Statistical Analysis** |
| | | (*b*) Describe any methods used to examine subgroups and interactions | 7-8 | **Materials and Methods, Statistical Analysis:** Furthermore, a secondary analysis, adjusted for confounders, was performed to gain insight into which subgroups of HCU were visited the most. |
| | | (*c*) Explain how missing data were addressed | 7-8 | **Materials and Methods, Statistical Analysis**: To indicate whether values were missing completely at random (MCAR) or missing at random (MAR), significant differences in baseline characteristics between participants with and without missing values at baseline were tested using independent T-tests for continuous values and chi-squared tests for categorical values. If significantly different, values were considered MAR, and imputation was conducted to reduce bias. Multiple imputation with fully conditional specification was used. A total of 10 different imputed datasets were generated. Ultimately, the imputed sets of parameter estimates were pooled using Rubin’s rules of combination. |
| | | (*d*) *Cohort study*—If applicable, explain how loss to follow-up was addressed<br><br>*Case-control study*—If applicable, explain how the matching of cases and controls was addressed | N/A | |

| | | | | |
|---|---|---|---|---|
| | | *Cross-sectional study*—If applicable, describe analytical methods taking account of sampling strategy | | |
| | | (*e*) Describe any sensitivity analyses | 7-8 | **Materials and Methods, Statistical Analysis:** Next, a sensitivity analysis was conducted to examine assumptions of the missing data by analyzing complete cases only. |
| **Results** | | | | |
| Participants | 13* | (a) Report numbers of individuals at each stage of study—eg numbers potentially eligible, examined for eligibility, confirmed eligible, included in the study, completing follow-up, and analysed | 8 | **Results:** Of the 1002 participants included in the CHECK cohort, 861 met the inclusion criteria for the current study. A total of 120 participants dropped out during the study. The reason for dropping out was unknown. The average follow-up time was 5.4 years. |
| | | (b) Give reasons for non-participation at each stage | 8 | **Results:** The reason for dropping out was unknown. |
| | | (c) Consider use of a flow diagram | N/A | N/A |
| Descriptive data | 14* | (a) Give characteristics of study participants (eg demographic, clinical, social) and information on exposures and potential confounders | 8-9 | **Results**: Baseline characteristics of the study population are presented in Table 1. |
| | | (b) Indicate number of participants with missing data for each variable of interest | 8 | **Results**: At baseline, PCI subscales had between 2.6% and 2.9% missing values. Over the years, HCU had missing data of 2.1% at baseline to 15% at T10. Of all values, 8.8% were missing and tests showed that the values were MAR. |
| | | (c) *Cohort study*—Summarise follow-up time (eg, average and total amount) | 8 | **Results**: The average follow-up time was 5.4 years. |

| | | | | |
|---|---|---|---|---|
| Outcome data | 15* | *Cohort study*—Report numbers of outcome events or summary measures over time | 8-13 | **Results, change in pain intensity and health status, course of healthcare use** |
| | | *Case-control study*—Report numbers in each exposure category, or summary measures of exposure | N/A | N/A |
| | | *Cross-sectional study*—Report numbers of outcome events or summary measures | N/A | N/A |
| Main results | 16 | (*a*) Give unadjusted estimates and, if applicable, confounder-adjusted estimates and their precision (eg, 95% confidence interval). Make clear which confounders were adjusted for and why they were included | 12-13 | **Results, relationship of pain coping style and healthcare use:** The analysis showed that the following variables interfered with the relationship between pain coping style and the course of the number of used healthcare services over 10 years: location of OA, NRS pain now, and NRS pain past week, all WOMAC subscales, and all subscales of the SF-36, except the subscale of general health. The results of the GEE analysis on the relationship between pain coping style in an early stage of OA and the course of the number of used healthcare services over 10 years, unadjusted and adjusted for confounders, are shown in Table 3. |
| | | (*b*) Report category boundaries when continuous variables were categorized | N/A | N/A |
| | | (*c*) If relevant, consider translating estimates of relative risk into absolute risk for a meaningful period | N/A | N/A |

Continued on next page

| | | | | |
|---|---|---|---|---|
| Other analyses | 17 | Report other analyses done—eg analyses of subgroups and interactions, and sensitivity analyses | 12-13 | **Results, relationship of pain coping style and healthcare use:** The sensitivity analysis showed no remarkable differences between imputed data and original data. The secondary analysis is shown in Table 4. |
| **Discussion** | | | | |
| Key results | 18 | Summarise key results with reference to study objectives | 13-14 | **Discussion:** This study aimed to examine the relationship between pain coping style in an early stage of OA and healthcare use over 10 years in patients with knee and/or hip OA. Results showed that patients with an active pain coping style use more different healthcare services over 10 years, compared to patients having a passive pain coping style. These results are independent of the change in pain and functioning over time as we adjusted for these confounders [...]. A surprising characteristic of the participants in the CHECK cohort was the large percentage (86.5%) of people with an active pain coping style at baseline [...]. Furthermore, results showed that active copers in this study did not show better health outcomes than passive copers at any time point [...]. An interesting finding in our study is that primary care services were used the most of all healthcare services. |
| Limitations | 19 | Discuss limitations of the study, taking into account sources of potential bias or imprecision. Discuss both direction and magnitude of any potential bias | 14-15 | **Discussion**: There are also some limitations to this study. First, the HCU-questionnaire only determined whether or not participants used prespecified healthcare services, and not how many times they used the services. Consequently, a higher HCU in this study cannot be interpreted as a higher amount of healthcare costs. Also, it may be argued that patients with a passive pain coping style tend to return to the same healthcare professional as opposed to patients with an active pain coping style, who might seek multiple sources of help. This could have led to the observation of less HCU in participants with a passive pain coping style. Second, self-reported healthcare use in patients with OA is often underreported, providing no accurate information. For future cohort studies, we recommend measuring HCU over the entire period and |

| | | | | |
|---|---|---|---|---|
| | | | | including the volume as well. To achieve an accurate and unbiased representation of HCU, retrospective cost diaries in combination with patients' medical files or administrative databases of healthcare insurances can be used. Third, the confidence interval of the results is wide, despite the large sample. This suggests a high dispersion in the data and represents the uncertainty in a generalization. Nevertheless, data show us results we should not ignore, since they indicate a potentially large impact on the healthcare system. In addition, these results are confirmed in other studies as well. At last, the content of the treatments was unknown. Therefore, we cannot determine which element of a given treatment may have contributed to an increase or reduction in used healthcare services per person. |
| Interpretation | 20 | Give a cautious overall interpretation of results considering objectives, limitations, multiplicity of analyses, results from similar studies, and other relevant evidence | 15 | In conclusion, the results of this study show that patients with early knee and/or hip OA with an active pain coping style use significantly more different healthcare services over 10 years, as opposed to those with a passive pain coping style. Further research is necessary to examine whether focusing on self-management skills as part of a treatment in people with an active coping style leads to a reduction in the number of used healthcare services and additional costs in the long term. |
| Generalisability | 21 | Discuss the generalisability (external validity) of the study results | 15 | This may have led to a biased sample, which may have affected the external validity of the results […]. Third, the confidence intervals of the results are wide, despite the large sample. This suggests a high dispersion in the data and represents the uncertainty in a generalization. |
| **Other information** | | | | |
| Funding | 22 | Give the source of funding and the role of the funders for the present study and, if applicable, for the original study on which the present article is based | 16 | **Role of funding source:** No funding was received from any bodies in the public, commercial, or not-for-profit sectors to carry out the work described in this article. |

*Give information separately for cases and controls in case-control studies and, if applicable, for exposed and unexposed groups in cohort and cross-sectional studies.

**Note:** An Explanation and Elaboration article discusses each checklist item and gives methodological background and published examples of transparent reporting. The STROBE checklist is best used in conjunction with this article (freely available on the Web sites of PLoS Medicine at http://www.plosmedicine.org/, Annals of Internal Medicine at http://www.annals.org/, and Epidemiology at http://www.epidem.com/). Information on the STROBE Initiative is available at www.strobe-statement.org.

## Table S2. Subgroups of healthcare use.

| Subgroup | Healthcare service based on the HCU-questionnaire |
|---|---|
| Self-care | Aspirin<br>Family/household help<br>Paracetamol<br>Pastor/priest/spiritual leader |
| NSAIDs | Ibuprofen, diclofenac (Voltaren), naproxine (Naproxen), celecoxib (Celebrex), refecobix (Vioxx)<br>Other drugs |
| Primary care | Family doctor<br>Physiotherapist<br>Psychologist<br>Exercise therapist, Cesar/Mensendieck<br>Occupational therapist<br>Social work<br>Other caregivers |
| Secondary care | Nurse/home care<br>Rehabilitation doctor<br>Rheumatologist<br>Orthopedist<br>Other specialist |
| Work-related care | Company doctor/Human Recourses<br>GMD / Gak / CWI / ABP / USZO (UWV) |

Abbreviations: NSAIDs = nonsteroidal anti-inflammatory drugs; GMD = Global Medical File; Gak = Joint Administration Office; CWI = Center for Work and Income; ABP = national civil pension fund; USZO (UWV) = institute employee insurance.
